# Inhibitory Effect of Astragalus Polysaccharides on Lipopolysaccharide-Induced TNF-α and IL-1β Production in THP-1 Cells

**DOI:** 10.3390/molecules17033155

**Published:** 2012-03-12

**Authors:** Xiaojuan He, Jun Shu, Li Xu, Cheng Lu, Aiping Lu

**Affiliations:** 1 Institute of Basic Research in Clinical Medicine, China Academy of Chinese Medical Sciences, Beijing 100700, China; 2 Institute of Clinical Medical Sciences, China-Japan Friendship Hospital, Beijing 100029, China; 3 College of Life Science and Technology, Beijing University of Chemical Technology, Beijing 100029, China; 4 School of Chinese Medicine, Hong Kong Baptist University, Kowloon Tong, Hong Kong, China

**Keywords:** Astragalus polysaccharides, anti-inflammatory, macrophage, MAPK, NF-κB

## Abstract

Astragalus polysaccharides (APS), one of main bioactive components in *Astragalus membranaceus* Bunge, has been reported to possess anti-inflammatory activities, but the molecular mechanisms behind this activity are largely unknown. This study aimed to investigate expression of inflammatory cytokines and the MAPK/NF-κB pathway in human THP-1 macrophages induced by lipopolysaccharide (LPS). The results showed that the concentrations of TNF-α and IL-1β released from LPS stimulated THP-1 cells increased significantly compared to control (*p* < 0.01). After treatment with APS, the TNF-α and IL-1β levels were significantly lower than those in the LPS group (*p* < 0.05). The mRNA expression of TNF-α and IL-1β were also inhibited. Mechanistic studies indicated that APS strongly suppressed NF-κB activation and down-regulated the phosphorylation of ERK and JNK, which are important signaling pathways involved in the production of TNF-α and IL-1β, demonstrating that APS could suppress the production of TNF-α and IL-1β by LPS stimulated macrophages by inhibiting NF-κB activation and ERK and JNK phosphorylation.

## 1. Introduction

Inflammation plays an important role in the progression of many diseases, including cardiovascular diseases, cancer and autoimmune diseases, such as rheumatoid arthritis (RA) [1,2,3,4,5]. These diseases are slowly disabling or fatal and affect a high percentage of the population. Inhibiting inflammatory response has become one focus of treating these diseases. Many researchers have demonstrated that macrophages, the key inflammatory cells, are closely associated with the pathologic process of inflammation [6,7]. They have three major functions in inflammatory response: antigen presentation, phagocytosis, and immunomodulation through production of various cytokines and growth factors, such as TNF-α and IL-1β [8].

Many traditional Chinese medicines (TCM) have been confirmed with anti-inflammatory activities [9,10,11]. *Astragalus membranaceus* Bunge (Leguminosae) is one of the most well-known herbal medicines widely used in China [12]. Its extracts contain numerous active ingredients, among which polysaccharides are one of the most important bioactive components. Apart from its immunomodulatory and hypoglycemic activities, several studies have revealed that Astragalus polysaccharides (APS) can be used for treating inflammatory diseases, including intestinal inflammation and RA [13,14]. It was able to reduce cell accumulation, swelling and arthritic index of the joints and serum concentrations of inflammatory mediators in adjuvant arthritic (AA) rats.

However, the mechanism(s) behind the anti-inflammatory effects of APS are still not elucidated. Since macrophages and their major producers of TNF-α and IL-1β play important roles in occurrence and development of inflammation, in this study we therefore explored the expression of inflammatory cytokines and NF-κB activation and phosphorylation of ERK, JNK in human THP-1 macrophages induced by lipopolysaccharide (LPS) in order to illuminate the initial mechanisms of the anti-inflammatory effects of APS.

## 2. Results and Discussion

### 2.1. Effects of APS on THP-1 Cell Viability

**Figure 1:**
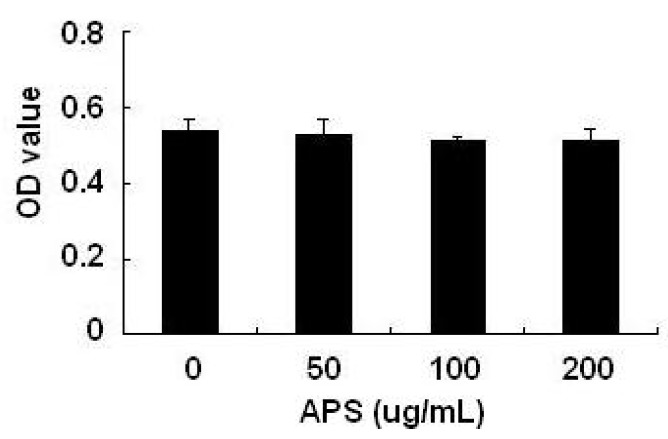
Effects of APS on THP-1 cell viability. A MTT assay was used to detect THP-1 cell viability after treatment with different concentration of APS (0, 50, 100 and 200 μg/mL). All experiments were performed three times. Data are the means ± S.D. of three independent experiments.

As shown in Figure 1, using the MTT assay, it was found that 50–200 μg/mL APS did not display any cellular toxicity against THP-1 cells over 24 h, which allows us to exclude a nonspecific cytotoxicity as a possible explanation for the decreased cytokines output.

### 2.2. Effect of APS on LPS-Induced Cytokine Expression in THP-1 Cells

**Figure 2:**
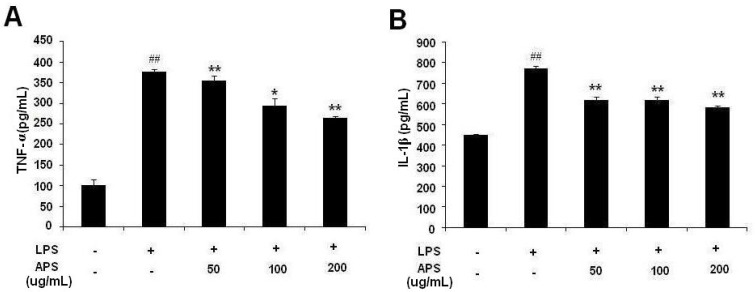
APS inhibited LPS-induced TNF-α and IL-1β production in THP-1 cells. (**A**) Level of TNF-α in culture supernatant of THP-1 cells; (**B**) Level of IL-1β in culture supernatant of THP-1 cells. Data are the means ± S.D. of three independent experiments. * *p* < 0.05, ** *p* < 0.01, *vs.* LPS group; ^## ^*p* < 0.01, *vs*. blank control group.

As shown in Figure 2, expression of TNF-α and IL-1β in the supernatant of LPS-stimulated THP-1 cells were significantly increased compared with the control group (*p* < 0.01). Different concentrations of APS could lower the level of TNF-α and IL-1β, and there was a significant difference compared with the LPS group (*p* < 0.05, *p* < 0.01).

**Figure 3:**
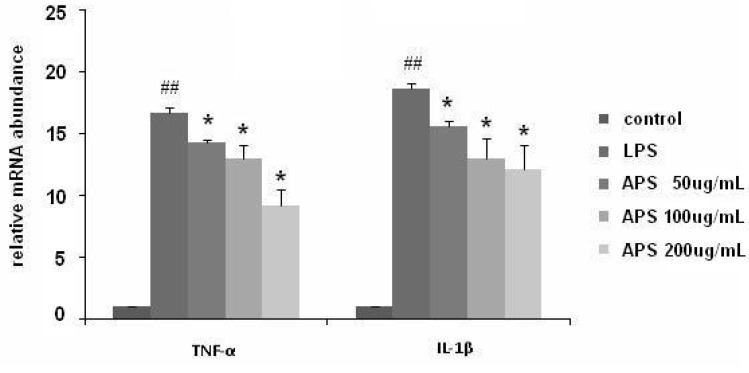
APS inhibited the mRNA expression of TNF-α and IL-1β in LPS-stimulated THP-1 cells. TNF-α and IL-1β mRNA expressions were determined using real time-PCR. GAPDH was used as the normal control. All experiments were performed three times. * *p* < 0.05, *vs*. LPS group; ^## ^*p* < 0.01, *vs*. blank control group.

To further detect the changes of TNF-α and IL-1β in LPS-stimulated THP-1 cells, real-time PCR was used. As shown in Figure 3, TNF-α and IL-1β mRNA expression in THP-1 cells was markedly increased upon exposure to LPS alone (*p* < 0.01). Treatment with APS remarkably inhibited LPS-induced mRNA expression of TNF-α and IL-1β (*p* < 0.05).

### 2.3. Effect of APS on NF-κB Activation in LPS-Stimulated THP-1 Cells

**Figure 4:**
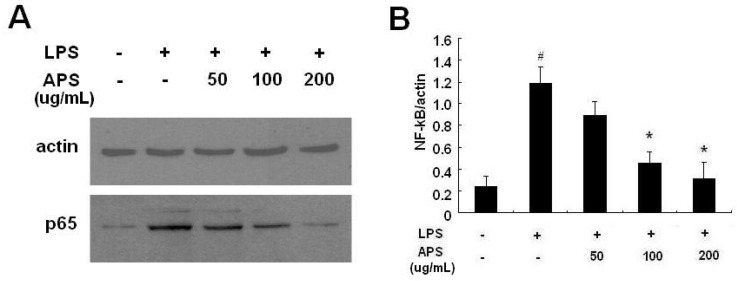
APS inhibited LPS-induced NF-κB expression in THP-1 cells. THP-1 cells were treated with LPS (1 μg/mL) and different concentration of APS (0, 50, 100 and 200 μg/mL) for 24 h at 37 °C. The cells were then collected and extracted for the detection of total forms of NF-κB p65 subunit by Western blot. (**A**) a representative Western blot result out of three experiments; (**B**) means ± S.D. of three independent experiments. * *p* < 0.05, *vs*. LPS group; ^# ^*p* < 0.05, *vs*. blank control group.

NF-κB is an important transcription factor involved in TNF-α and IL-1β production in LPS-stimulated macrophages. To further understand the mechanism of APS-mediated anti-inflammatory effects in THP-1 cells, we next examined whether APS had an inhibitory effect on the expression of NF-κB. As shown in Figure 4, the NF-κB (p65) level was significantly up-regulated in LPS-stimulated THP-1 cells. Treatment with APS could remarkably down-regulate NF-κB levels.

### 2.4. Effect of APS on Phosphorylation of ERK and JNK in LPS-Stimulated THP-1 Cells

**Figure 5:**
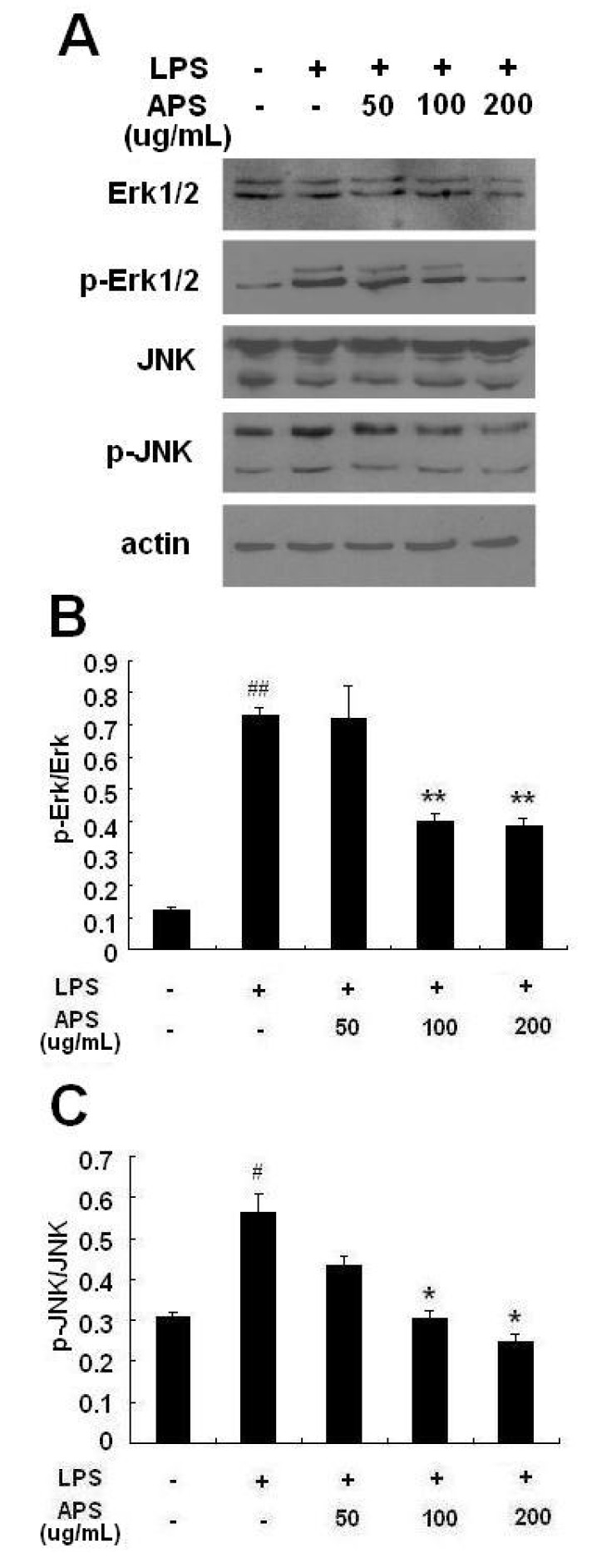
APS inhibited phosphorylation of MAPKs in LPS-stimulated THP-1 cells. THP-1 cells were treated with LPS (1 μg/mL) and different concentration of APS (0, 50, 100 and 200 μg/mL) for 24 h at 37 °C. The cells were then collected and extracted. Western blot analysis was performed to detect p-ERK, total ERK, p-JNK, total JNK and β-actin. (**A**) Western blot of the MAPKs isoforms: A representative result out of three experiments; (**B**) relative ratio of p-ERK/ERK; (**C**) relative ratio of p-JNK/JNK. * *p *< 0.05, ** *p* < 0.01, *vs*. LPS group; ^# ^*p* < 0.05, ^## ^*p* < 0.01, *vs*. blank control group. Data are presented as mean ± S.D., and the data shown are representative of three independent experiments.

To determine whether MAPK signaling pathways are involved in the anti-inflammatory effects of APS, we investigated the phosphorylation of two MAPK signaling molecules, ERK1/2 and JNK, by Western blot. As shown in Figure 5, the levels of phosphorylated ERK1/2 and JNK were remarkably increased in THP-1 cells stimulated with LPS. APS treatment decreased LPS-induced ERK1/2 and JNK phosphorylation in a concentration-dependent manner. However, total levels of ERK and JNK did not significantly change among these groups.

### 2.5. Discussion

Astragalus polysaccharides (APS), extracted from *Astragalus membranaceus* Bunge (Leguminosae), possess multiple pharmacological and immunomodulatory activities. They play a role in disease therapy by regulating the functions of immune cells and expression of cytokines, increasing activity of antioxidant enzymes, and reducing lipid peroxidation, *etc*. [13,15,16]. In this study, we report that APS can regulate the function of LPS-stimulated THP-1 cells, a good macrophage model system [17]. Our results suggest that APS can inhibit production of inflammatory cytokines by THP-1 cells through MAPK/NF-κB pathway, which may be the mechanism of the anti-inflammatory effect of APS.

Macrophages play a critical role in the initiation, maintenance, and resolution of inflammation. Stimulated by bacterial endotoxins, e.g., lipopolysaccharide (LPS), macrophages can secrete large amounts of inflammatory cytokines, such as TNF-α and IL-1β, and these inflammatory cytokines further activate macrophages, creating a vicious cycle and therefore increasing the inflammatory response. Consequently, therapeutic interventions targeting macrophages and their products have become a focal point for controlling inflammatory diseases. Examples of such therapeutics include targeting anti-inflammatory drug to macrophages [18], and using inflammatory cytokine antagonists, such as IL-1-receptor antagonist (anakinra) and TNF antagonists (infliximab, etanercept and adalimumab) [19,20,21]. Despite their remarkable success, the cost and numerous adverse effects still limit their application [22,23,24,25]. Compared with these chemical and biological drugs, some Traditional Chinese Medicines (TCMs) from a wide variety of sources show relatively low toxicity and good efficacy in this area. In this study, we demonstrated that APS, a cheap and low-toxicity TCM, could effectively inhibit inflammatory cytokine production by THP-1 cells.

NF-κB is a major transcription factor that regulates the genes responsible for both the innate and adaptive immune response. Incorrect regulation of NF-κB has been linked to cancer, inflammatory and autoimmune diseases, *etc*. NF-κB has been proved to be a key player in the inflammatory response. It induced gene expression of inflammatory cytokines such as TNF-α and IL-1β. In our experiment, APS could remarkably down-regulate NF-κB levels in LPS-stimulated THP-1 cells, which demonstrated that APS had a potential role on the suppression of the NF-κB signaling pathway in the inflammatory response-mediated TNF-α and IL-1β production. Since NF-κB activation is induced by the dissociation of IκBα and IκBβ, which are phosphorylated by IκB kinases, the amount of degradation of IκBα and IκBβ is commonly used as an indicator to confirm the activation of NF-κB [26]. So whether APS influences IκB needs to be further examined.

MAPKs are a family of serine/threonine protein kinases responsible for most cellular responses to cytokines and crucial for regulation of the production of inflammation mediators [27]. A few natural extracts have been shown to inhibit the expression of proinflammatory genes by regulating the phosphorylation of MAPK pathways [28,29,30]. To investigate whether these pathways are involved in the molecular mechanism(s) of TNF-α and IL-1β inhibition of APS in THP-1 cells, we explored the levels of phosphorylation of ERK and JNK. The levels of phosphorylation of ERK and JNK were up-regulated in THP-1 cells induced by LPS, but remarkably down-regulated by APS. These results implied that APS might affect the MAPK signaling cascade. In this study, we did not explore how APS influenced the MAPK/NF-κB signal pathway. As well known, tumor necrosis factor receptor-associated factor 6 (TRAF6) functions as a signal transducer in the NF-κB pathway that activates IκB kinase (IKK) in response to proinflammatory cytokines [31]. Transforming growth factor β-activated kinase-1 (TAK1) is a member of the MAPK kinase kinase (MAPKKK) family that mediates MAPK and IKK activation via interaction with TRAF6 [32,33,34]. Therefore, it is worthy of further study to confirm whether APS affects the MAPK/NF-κB pathway through regulation of TRAF6-TAK1 signalling complexes.

## 3. Experimental

### 3.1. Chemicals and Reagents

Astragalus Polysaccharides (APS) of about 98% purity was purchased from Pharmagenesis, Inc. (Redwood City, CA, USA). There was no detectable level of endotoxin (<0.10 endotoxin units/mL) in the APS samples by the assay of Endospecy. Phorbol 12-myristate 13-acetate (PMA) and lipopolysaccharide (LPS) were purchased from Sigma-Aldrich (St. Louis, MO, USA). Anti-human TNF-α ELISA kit and anti-human IL-1β ELISA kit were purchased from eBioscience (San Diego, CA, USA). HiFi-MMLVcDNA kit and UltraSYBR Mixture were purchased from CWbio Co. Ltd. (Beijing, China). Anti-human NF-κB (p65), p-SAPK/JNK, SAPK/JNK, p-ERK1/2 and ERK1/2 antibodies were purchased from Cell Signaling Technology (Danvers, MA, USA). Horseradish peroxidase (HRP)-labeled anti-rabbit IgG (H+L) antibody was purchased from Santa Cruz Biotechnologies (Santa Cruz, CA, USA).

### 3.2. Cell Culture

THP-1, human monocyte-like cells, were obtained from the cell culture center of the Chinese Academy of Medical Sciences and cultured in RPMI1640 medium supplemented with 10% heat-inactivated fetal calf serum (FCS), 100 U/mL penicillin, and 100 μg/mL streptomycin.

### 3.3. MTT Assay for THP-1 Cells Viability

THP-1 cells (1 × 105 cells/mL) were seeded in 96-well plate and induced by PMA (10 ng/mL) for 48 h in a 37 °C, 5% CO_2_ incubator. Then, different concentrations of APS were added. Twenty four hours later, MTT (5 mg/mL) was added and incubated for an additional 4 h. The supernatants in each well were carefully removed, and 100 μL DMSO was then added into each well, followed by incubation at 37 °C for 10 min with gentle shaking. The absorbance at 570 nm was measured with a microplate reader.

### 3.4. Macrophage Stimulation

THP-1 cells were pre-treated with PMA (10 ng/mL) for 48 h to induce macrophages. Then, they were stimulated with LPS (1 μg/mL) with or without different concentrations of APS for 24 h at 37 °C in presence of 5% CO_2_. Cells were collected for real-time PCR and Western blot, and culture supernatants were used for enzyme-linked immunosorbent assay (ELISA).

### 3.5. Real-Time PCR

Total RNA was extracted and dissolved in RNA-free water and quantified using UV-clear microplates. Then, single-strand cDNA was synthesized from 2 μg total RNA by using HiFi-MMLVcDNA kit. Real-Time PCR was performed using Qiagen Rotor Qgene and RealSYBR Mixture Commercial kits. GAPDH was used as the reference gene. The primers used were as follows: IL-1β, sense, 5'-TACAAGGAGAAGAAAGTAATGACAA-3', antisense, 5'-AGCTTGTTATTGATTTCTATCTTGT-3'; TNF-α, sense, 5'-CTCCTCACCCACACCATCAGCCGCA-3', antisense, 5'-ATAGATGGGCTCATACCAGGGCTTG-3'; GAPDH, sense, 5'-CTCATGACCACAGTCCATGC-3', antisense, 5'-CACATTGGGGGTAGGAACAC-3'. The cycling conditions were 95 °C for 10 min, followed by 40 cycles of 95 °C for 15 s and 60 °C for 60 s. All samples were measured in triplicate. Differences in gene expression were calculated using the 2-cycle threshold method.

### 3.6. Cytokine Assays

Levels of IL-1β and TNF-α in culture supernatants were detected by ELISA kits according to the manufacturers’ instructions.

### 3.7. Western Blot

Cells were lysed with ice-cold cell lysis buffer. Protein concentration was then determined using BCA protein assay kit. Samples of cell lysates were separated by 10% SDS-PAGE and then transferred onto nitrocellulose membranes. After being placed in blocking buffer, the membranes were incubated with following primary antibodies (1:2,000 dilution): Anti-ERK, anti-JNK, anti-p-ERK, anti-p-JNK, and anti-p65. Then, peroxidase-conjugated secondary antibodies were used. The protein bands were visualized by the ECL kit. The intensities of the protein bands were analyzed by Gel-Pro 3.2 software. β-actin protein was used as the internal control.

### 3.8. Statistical Analysis

Data were presented as mean ± S.D. Differences were evaluated using Statistical Package for Social Science 11.0 (SPSS11.0). Statistical analysis was performed using One-way ANOVA. *P* < 0.05 was considered to be statistically significant.

## 4. Conclusions

In conclusion, our results indicated that APS could lower the production of TNF-α and IL-1β secreted by LPS stimulated THP-1 cells, which might occur through inhibition of the MAPK/NF-κB signaling pathway.
